# Anterior Arch Fracture of the Atlas on Postoperative Day 6 Following Unilateral C1 Laminectomy Requiring Occiput-to-C2 Fusion: A Case Report

**DOI:** 10.7759/cureus.113373

**Published:** 2026-07-25

**Authors:** Nobuaki Naito, Takanobu Miyamoto, Hokuto Yamashita, Shigeo Ueda, Atsuhiko Toyoshima

**Affiliations:** 1 Neurosurgery, Shinaikai Spine Center, Katano Hospital, Katano, JPN; 2 Neurosurgery, Wakayama Medical University, Wakayama, JPN; 3 Neurological Surgery, Shinaikai Spine Center, Katano Hospital, Katano, JPN

**Keywords:** anterior arch fracture, atlas fracture, c1 laminectomy, craniovertebral junction, occipitocervical fusion, occiput-to-c2 fixation

## Abstract

C1 laminectomy is a well-established posterior decompression procedure for craniovertebral junction stenosis. However, disruption of the ring structure of the atlas may alter load distribution across the anterior arch and lateral masses, occasionally resulting in atraumatic anterior arch fractures. Although several cases have been reported following bilateral C1 posterior arch resection or foramen magnum decompression, most developed weeks to months after surgery. In contrast, anterior arch fracture occurring during the acute postoperative period after unilateral C1 laminectomy has rarely been described. We report the case of an 82-year-old woman who developed an anterior arch fracture of the atlas on postoperative day 6 after left unilateral C1 laminectomy combined with C3-7 laminoplasty for cervical spinal canal stenosis. She presented with the sudden onset of severe headache. Initial head computed tomography (CT) showed no intracranial abnormalities. Because the headache persisted, cervical CT was obtained and demonstrated an anterior arch fracture of C1. Retrospective review revealed that the fracture had already been present on CT acquired on postoperative day 6. Conservative management with cervical immobilization was initiated; however, follow-up imaging demonstrated progressive fracture displacement and lateral mass subluxation, prompting surgical stabilization. Intraoperatively, marked instability of the C1 lateral masses precluded safe placement of C1 screws, and occiput-to-C2 fusion with autologous iliac crest bone grafting was performed. The patient's headache resolved promptly after surgery, and no neurological deterioration occurred. At the eight-month follow-up, progressive bony union was confirmed without recurrence of symptoms. This case represents one of the earliest reported anterior arch fractures following C1 laminectomy. The unusually early onset may have been related to a combination of asymmetric biomechanical loading after unilateral posterior arch resection, age-related impairment of bone quality, and additional biomechanical changes associated with multilevel laminoplasty, although a causal relationship cannot be established from a single case. Importantly, postoperative headache following C1 decompression should not be attributed solely to intracranial pathology or cerebrospinal fluid-related disorders. When new-onset headache or occipital pain develops after C1 laminectomy, early cervical CT should be considered to exclude structural complications. In patients with progressive fracture displacement or significant C1 instability, rigid internal fixation, including occiput-to-C2 fusion, may provide reliable stabilization.

## Introduction

C1 posterior arch resection is a well-established decompressive procedure for craniovertebral junction stenosis caused by degenerative disorders or Chiari malformation in the absence of instability. However, resection of the posterior arch disrupts the ring structure of the atlas and alters load transmission across the C1 ring. Biomechanical studies have suggested that loss of posterior support increases stress concentration on the anterior arch and lateral masses, occasionally resulting in anterior arch fractures [1].

Iatrogenic anterior arch fractures following C1 posterior arch resection are uncommon, and only isolated case reports and small case series have been published to date. Although their incidence, natural history, and optimal management remain incompletely understood, these fractures are clinically important because they may occur in the absence of trauma and often present weeks to months after surgery [2,3]. Patients typically present with severe occipital pain, and conservative treatment may fail because of progressive fracture displacement or C1 instability, ultimately requiring surgical stabilization [4]. However, evidence regarding anterior arch fractures occurring during the acute postoperative period following unilateral C1 posterior arch resection remains extremely limited. In particular, the potential contribution of asymmetric biomechanical loading associated with unilateral decompression remains poorly elucidated.

We report the case of an 82-year-old woman who developed severe headache on postoperative day 6 following left unilateral C1 posterior arch resection performed for craniovertebral junction stenosis caused by degenerative disease. Further evaluation revealed a right anterior arch fracture of the atlas with lateral mass dissociation. Because external immobilization failed to prevent progressive instability, occiput-to-C2 fusion was ultimately required. This case highlights one of the earliest reported postoperative anterior arch fractures following unilateral C1 posterior arch resection and provides further clinical insight into this rare but potentially serious complication.

## Case presentation

An 82-year-old woman presented with numbness of the right upper extremity and intermittent episodes of bilateral lower extremity weakness. Her medical history included lumbar spinal canal stenosis and osteoporotic lumbar vertebral compression fractures. She had not undergone formal evaluation of bone mineral density before surgery and had no history of osteoporosis treatment or long-term corticosteroid use. Neurological examination demonstrated cervical myelopathy with moderate gait disturbance, bilateral lower extremity weakness, hyperreflexia, and right upper extremity sensory disturbance.

**Figure 1:**
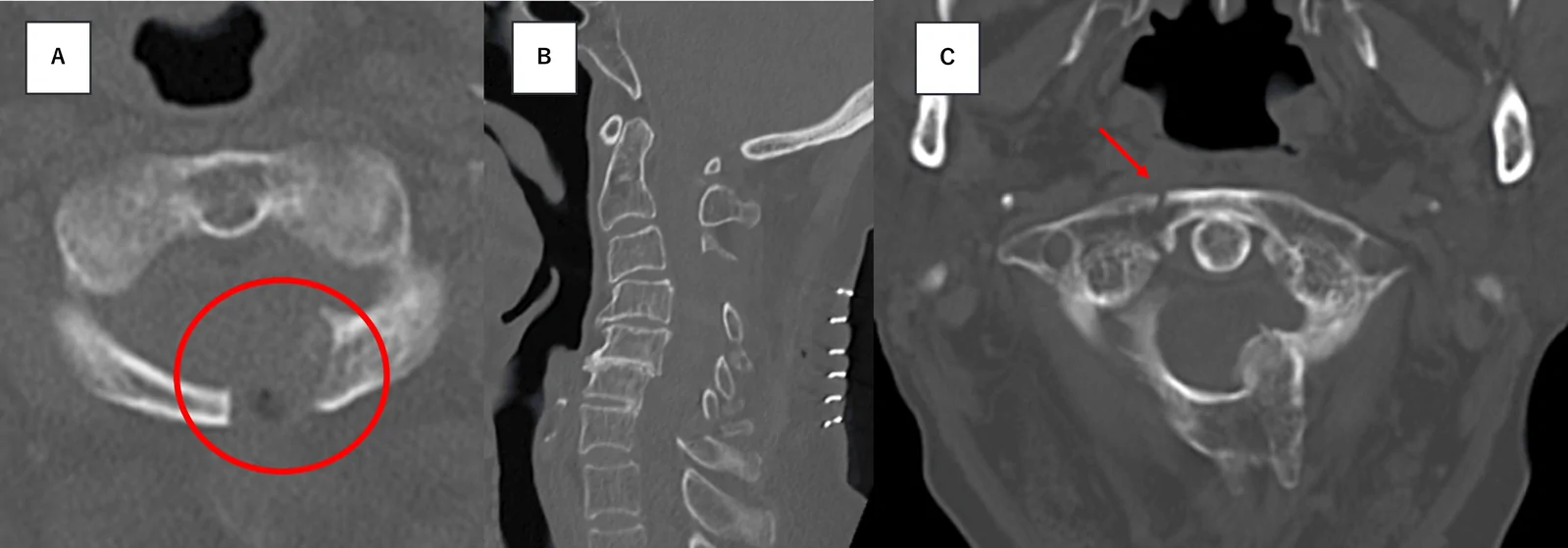
Preoperative and early postoperative imaging findings (A) Axial CT image following left unilateral C1 laminectomy (10.49 mm resection width). (B) Sagittal CT image after surgery. (C) CT obtained on postoperative day 6 demonstrating right anterior arch fracture of C1 CT: computed tomography

Magnetic resonance imaging demonstrated compression at the craniovertebral junction caused by degenerative hypertrophy of the left posterior arch of C1 together with developmental cervical spinal canal stenosis from C3 to C7. The patient was diagnosed with cervical myelopathy secondary to cervical canal stenosis and underwent left unilateral C1 hemilaminectomy (resection width, 10.49 mm) combined with C3-7 laminoplasty (Figures 1A, 1B). Cervical computed tomography (CT) obtained on postoperative day 1 demonstrated no evidence of fracture.

On postoperative day 6, the patient suddenly developed headache. Because intracranial complications, including postoperative hemorrhage, were considered more likely than a cervical structural complication at that stage, head CT was performed as the initial investigation. No intracranial abnormalities were identified, and the patient was managed with close clinical observation.

At the patient's strong request, she was temporarily discharged on postoperative day 9 despite persistent headache. Because the headache did not improve, she returned to our hospital on postoperative day 12. Cervical CT demonstrated a right anterior arch fracture of the atlas. Retrospective review of the CT obtained on postoperative day 6 confirmed that the fracture had already been present at the onset of symptoms (Figure 1C).

**Figure 2:**
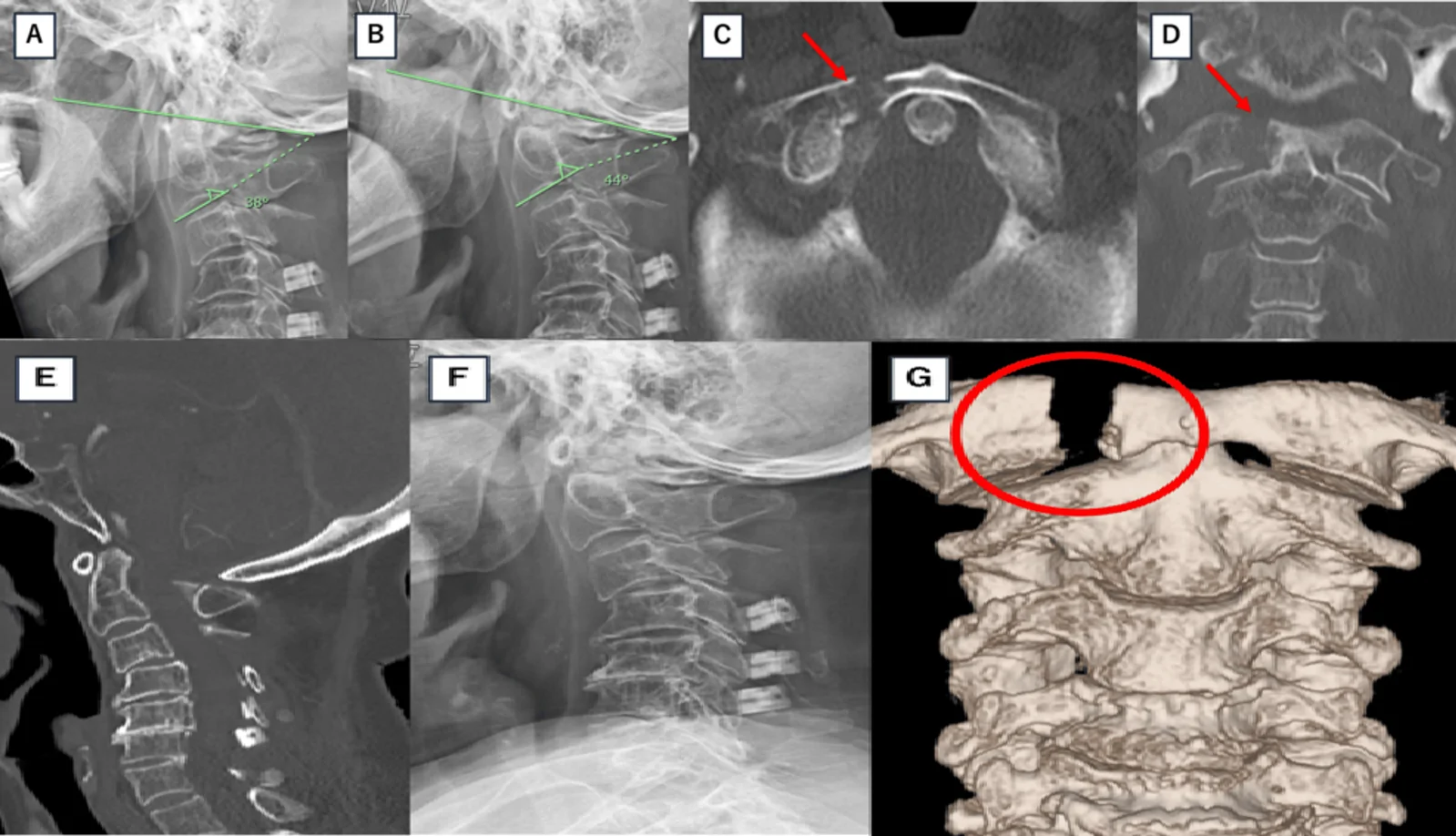
Progression of fracture displacement and lateral mass subluxation (A) Prefracture occiput-C2 angle measured at 38°. (B) Postfracture occiput-C2 angle increased to 44°. (C) Axial CT image demonstrating progression of anterior arch fracture. (D) Coronal CT image demonstrating lateral mass displacement. (E) Sagittal radiograph obtained during follow-up. (F) Lateral radiograph showing change in occiput-C2 alignment. (G) Three-dimensional reconstructed CT image CT: computed tomography

Initial treatment consisted of rigid cervical immobilization with a cervical collar and close radiographic follow-up. However, repeat evaluation two weeks later demonstrated progressive instability. Cervical radiographs showed that the occiput-C2 angle (McGregor line-C2 inferior endplate angle) had increased from 38° before fracture occurrence to 44° after fracture development, indicating deterioration of craniocervical alignment (Figures 2A, 2B). Cervical CT further demonstrated widening of the fracture gap and progressive lateral mass dissociation (Figures 2C-2G). Because fracture displacement and C1 instability progressed despite external immobilization, conservative treatment was considered insufficient, and surgical stabilization was recommended. The patient was therefore readmitted for operative treatment. To restore physiological craniocervical alignment, occiput-to-C2 fixation was planned using the prefracture occiput-C2 angle of 38° as the target alignment.

Surgical findings

The head was secured using a three-point fixation system, and the patient was placed in the prone position. Under fluoroscopic guidance, the occiput-C2 angle was adjusted to the planned 38°. A midline skin incision was made from the inion to approximately the C4 level. Adequate exposure was obtained between the occiput and C1-C2. However, C1 was unstable, and the right side was particularly mobile. Intraoperative CT was obtained using an O-Arm imaging system. Because of the marked instability of C1, placement of screws into C1 was considered infeasible, and occiput-C2 fixation was selected. Under navigation guidance, pilot holes were created bilaterally. Screws were inserted with minimal tapping at the entry point. Because C2 was also unstable, special attention was paid to rotational alignment. A 3.5 × 20 mm cortical screw was inserted into the right C2 pedicle and a 3.5 × 22 mm cortical screw into the left C2 pedicle. The screw insertion points in the occiput were then identified. Three 3.5 × 8 mm screws were inserted in the midline occiput, and one 3.5 × 6 mm screw was inserted on the right side. Rods were contoured to maintain the occiput-C2 angle and were secured between the occiput and C2. Subsequently, the iliac crest was exposed through a separate incision, and a rectangular corticocancellous bone graft measuring 15 × 20 mm was harvested. The harvested iliac bone graft was placed between the occiput and C2. Compression of the graft was achieved by connecting the rods, and fibrin glue was applied before closure.

**Figure 3:**
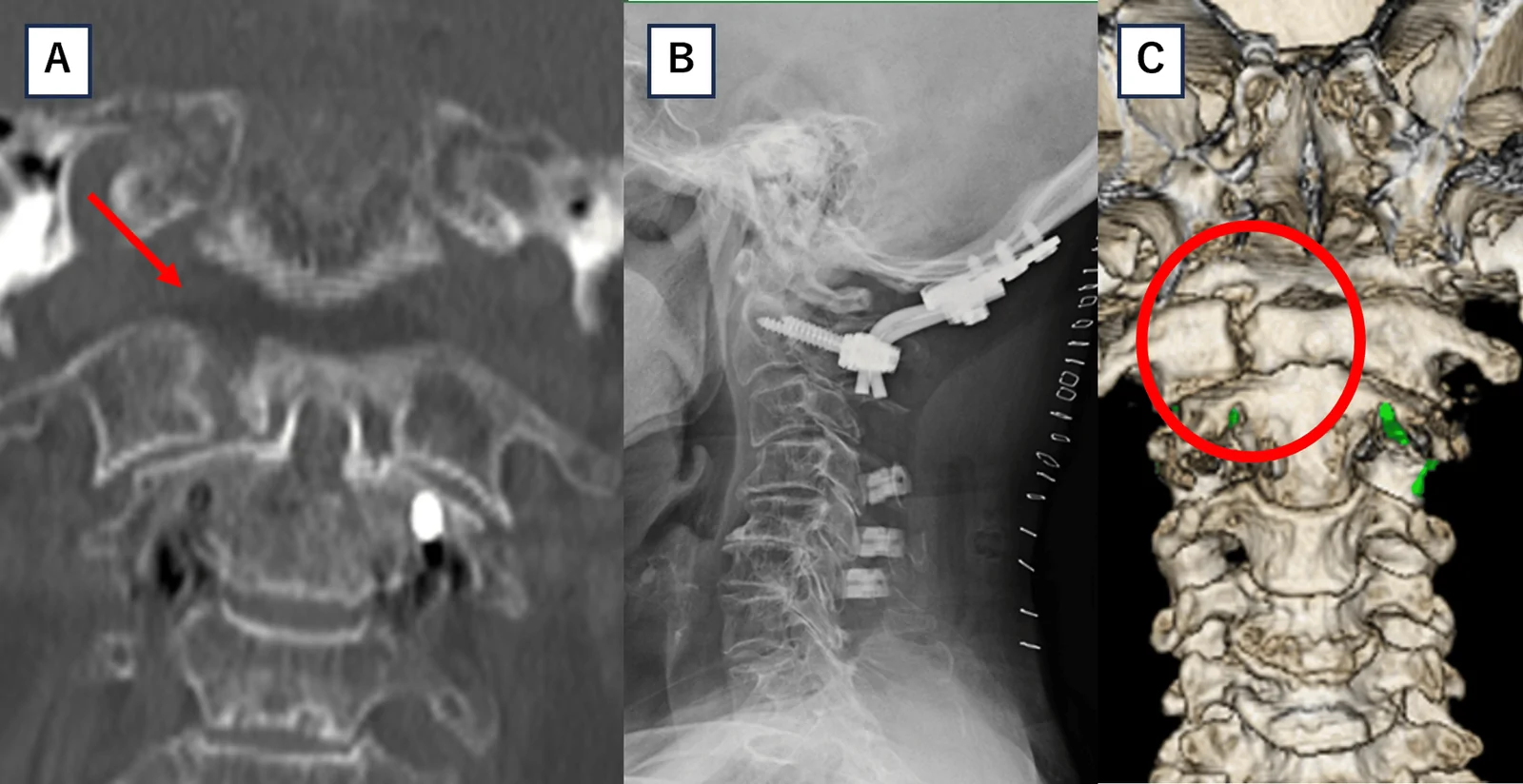
Postoperative imaging after occiput-to-C2 fixation (A) Coronal CT image after fixation. (B) Sagittal radiograph demonstrating restored alignment. (C) Three-dimensional reconstructed CT image after occiput-to-C2 fixation CT: computed tomography

Postoperatively, the occiput-C2 angle was restored to a configuration similar to that before fracture occurrence, and satisfactory fixation was achieved (Figures 3A-3C). The patient's headache improved, and no deterioration of neurological findings was observed. She was discharged home.

**Figure 4:**
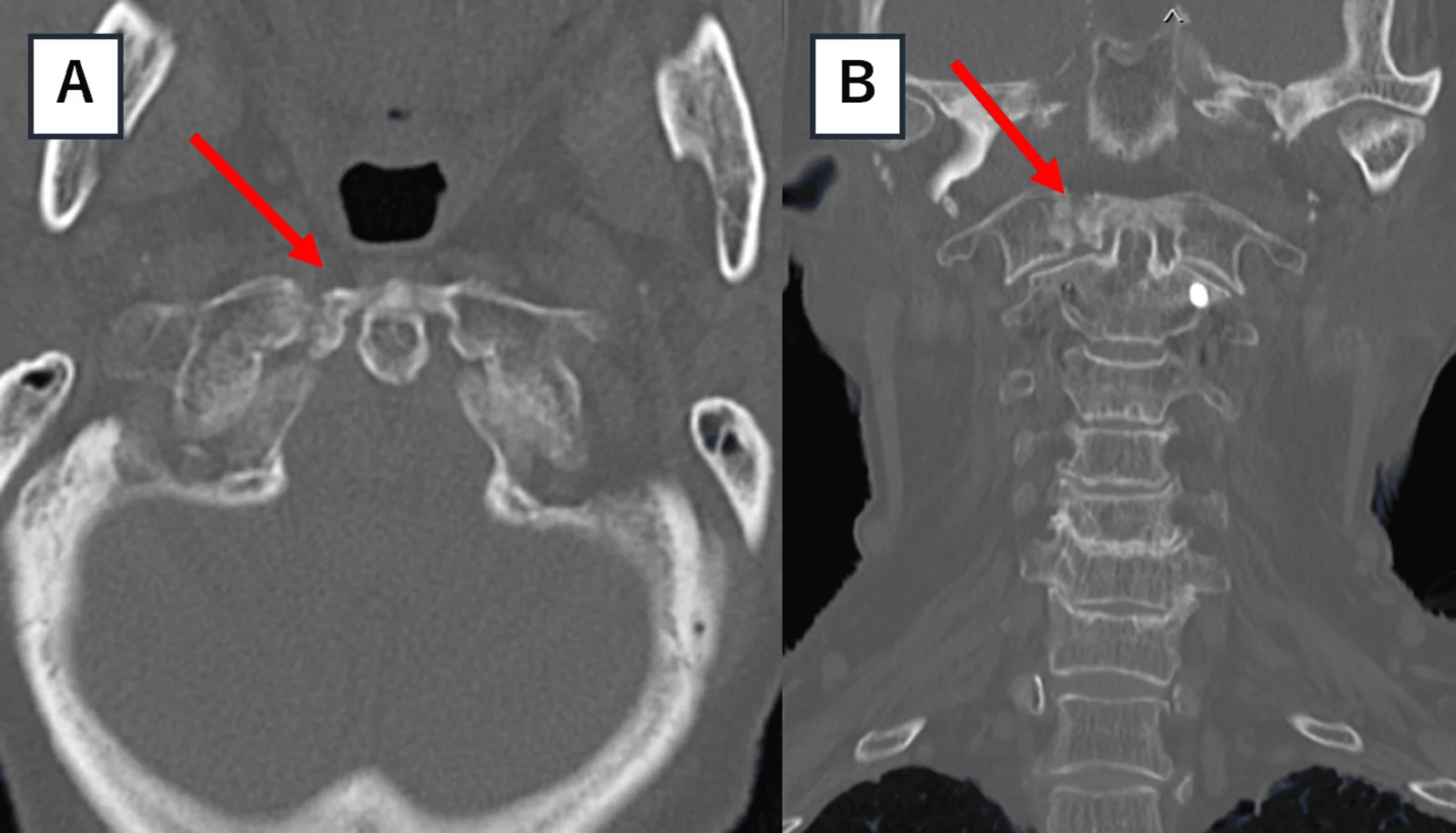
Follow-up imaging obtained nine months after occiput-to-C2 fixation (A) Axial CT image demonstrating progressive bone union. (B) Coronal CT image confirming maintained fixation and fusion progression

At eight months after surgery, cervical CT demonstrated progressive bone union, and no worsening of neurological symptoms was observed during follow-up (Figures 4A, 4B).

## Discussion

The principal finding of the present case is the exceptionally early occurrence of an anterior arch fracture, which became clinically apparent with headache only six days after unilateral C1 posterior arch resection. As summarized in Table 1, anterior arch fractures following C1 posterior arch resection or foramen magnum decompression (FMD) have been reported only sporadically, including the cases described by O'Shaughnessy et al. and Hirano et al. [5,6]. However, most previously reported cases occurred several weeks to months after surgery or following FMD for Chiari malformation [1-8]. In addition, the case reported by Matsunaga et al. developed after revision occipitocervical surgery, whereas the present case occurred after an initial decompression procedure. To our knowledge, reports describing anterior arch fractures occurring during the acute postoperative period after unilateral C1 posterior arch resection remain extremely limited. In contrast, the present patient developed symptoms on postoperative day 6, representing one of the earliest reported postoperative presentations and comparable only to the "several days" onset reported by Ishihama et al. (Table 1). This observation suggests that anterior arch fracture following C1 posterior arch resection should not be regarded solely as a delayed complication and may also occur during the acute postoperative period.

**Table 1:** Previously reported cases of anterior arch fracture FMD: foramen magnum decompression

Study	Year	Procedure	Onset	Treatment	Outcome
O'Shaughnessy et al. [5] (Case 1)	2004	FMD	5 months	C1-3 fixation	Bone union
O'Shaughnessy et al. [5] (Case 2)	2004	FMD	6 months	Halo vest	Bone union
Hirano et al. [6]	2011	FMD	7 months	C1-2 fixation	Union achieved
Sano et al. [9]	2020	FMD + C1 laminectomy	7 months	Goel-Harms fixation	Bone union
Palmieri et al. [10]	2021	C1 posterior arch procedures	Intraoperative	Posterior reconstruction	Fusion achieved
Kim et al. [4]	2022	C1 laminectomy	8 months	C1-2 fusion	Pain improved
Kawasaki et al. [1]	2023	C1 laminectomy + laminoplasty	1.5 years	Occipitocervical fusion	Stable recovery
Matsunaga et al. [7]	2024	Revision O-C fusion + C1 laminectomy	10 days	Halo vest	Symptom relief
Ishihama et al. [8]	2025	C1/C4-6 laminectomy	Several days	Cervical collar	Stable nonunion
Present case	2026	Unilateral C1 laminectomy	6 days	Occiput-C2 fixation	Improved

From a biomechanical perspective, the atlas forms a ring structure consisting of the anterior arch, posterior arch, and lateral masses, which distributes axial loads throughout the ring [11]. Resection of the posterior arch disrupts this ring structure, altering the normal load-distribution mechanism and increasing stress concentration on the anterior arch and lateral masses [2,3,10]. Furthermore, Shimizu et al. identified ankylotic changes below C2 as an independent risk factor for anterior arch fracture following C1 laminectomy [2].

A characteristic feature of the present case is that, whereas most previously reported cases occurred after bilateral posterior arch resection or FMD, this patient developed a right anterior arch fracture following left-sided unilateral hemilaminectomy. During C1 laminectomy, approximately 20 mm of the posterior arch is generally removed while preserving the lateral masses. In the present case, the resection width was only 10.49 mm, substantially smaller than the approximately 20 mm width commonly reported. This finding suggests that even limited unilateral decompression may be sufficient to alter the biomechanical environment of the atlas and contribute to fracture development. Because stenosis was confined to the left side, decompression was performed unilaterally [4]. Although unilateral decompression may partially preserve the integrity of the atlas ring, it may also result in asymmetric load distribution. One possible explanation for the fracture pattern observed in this case is that unilateral decompression altered load transmission across the atlas, leading to increased stress at the contralateral anterior arch-lateral mass junction. However, this remains a hypothesis based on the present case and existing biomechanical concepts, as no biomechanical studies have directly evaluated the relationship between unilateral C1 posterior arch resection and contralateral anterior arch fracture. Further biomechanical and clinical investigations are warranted to clarify this mechanism.

Although bone mineral density was not evaluated in this patient, she was an elderly woman with a history of lumbar vertebral compression fracture, suggesting that bone fragility may also have contributed to fracture development. In addition, biomechanical alterations associated with concomitant C3-7 laminoplasty may have further increased local stress on the anterior arch and lateral masses of C1, potentially contributing to the exceptionally early onset of fracture on postoperative day 6 [2,3,11]. Because this is a single case, the relative contribution of each of these factors cannot be determined, and fracture development was likely multifactorial.

The diagnostic challenges encountered in this case also provide important clinical insights. Anterior arch fractures frequently present with occipital pain or posterior neck pain in the absence of trauma [2,4,9]. However, because many reported cases have occurred in a delayed fashion, headache during the acute postoperative period is more commonly attributed to wound pain, tension-type headache, intracranial hypotension, or intracranial hemorrhage. Consequently, cervical osseous injury may not be considered during the initial evaluation, resulting in delayed diagnosis.

In the present case, intracranial pathology was initially suspected when the patient developed sudden headache, and head CT was therefore performed as the first diagnostic examination. Because cervical CT was not obtained at that time, the diagnosis of anterior arch fracture was delayed. Retrospective review confirmed that the fracture had already been present on postoperative day 6. Although plain radiographs have limited sensitivity for detecting subtle fractures and lateral mass displacement, CT enables detailed assessment of fracture morphology and associated instability [3,12]. Therefore, when patients who have undergone C1 posterior arch resection develop new-onset or rapidly worsening occipital pain or headache, clinicians should maintain a low threshold for obtaining cervical CT in addition to head CT during the initial evaluation, particularly when intracranial imaging is unrevealing [9,12].

Treatment strategy depends on the presence of instability as well as the degree of fracture separation and lateral mass displacement. Stable, nondisplaced fractures may be managed conservatively with rigid cervical collar or Halo vest immobilization [3]. However, despite rigid cervical collar immobilization, the present patient demonstrated progressive fracture separation and lateral mass dissociation. Once an anterior arch fracture develops following posterior arch resection, the atlas ring is effectively disrupted at two sites. Consequently, cervical collar immobilization alone may not provide sufficient stability to adequately control axial loading, rotational forces, and lateral bending moments in some patients.

The principal surgical options include C1-2 fixation and occiput-to-C2 fixation. C1-2 fixation, most commonly performed using the Goel-Harms technique, has the advantage of preserving a degree of atlantoaxial rotation [13,14]. However, in the present case, both preoperative CT and intraoperative findings demonstrated marked instability of the C1 lateral mass, precluding safe placement of C1 screws. Therefore, occiput-to-C2 fixation combined with autologous iliac crest bone grafting was selected to achieve stable fixation.

Although occiput-to-C2 fixation sacrifices upper cervical motion, it can provide reliable biomechanical stability and high fusion rates in patients with poor bone quality or severe craniocervical instability [8,15]. In addition, the occiput-C2 angle is an important parameter associated with postoperative dysphagia and airway stenosis [16,17]. In the present case, the prefracture occiput-C2 angle of 38° was reproduced, restoring physiological craniovertebral alignment while minimizing the risk of postoperative alignment-related complications.

Taken together, this case expands the limited literature on anterior arch fracture following C1 posterior arch resection by demonstrating that this rare complication may occur during the acute postoperative period, even after unilateral decompression. Although the proposed biomechanical mechanism remains hypothetical, the fracture pattern observed in the present case raises the possibility that asymmetric loading following unilateral posterior arch resection may contribute to fracture development. Clinicians should maintain a low threshold for considering anterior arch fracture when patients develop new-onset occipital pain or headache after C1 posterior arch resection. When clinical suspicion persists, cervical CT should be considered in addition to head CT, particularly when intracranial imaging is unrevealing. Early recognition may facilitate timely intervention before progressive instability develops.

Limitation

This study has several limitations. First, bone mineral density was not evaluated; therefore, the contribution of osteoporosis to fracture development could not be objectively assessed. Second, this report describes a single case and does not establish a causal relationship between unilateral C1 hemilaminectomy and contralateral anterior arch fracture. Third, the interpretation of the imaging findings obtained on postoperative day 6 is based on retrospective analysis. Further accumulation of cases and biomechanical investigations will be necessary to clarify the underlying mechanism and to establish preventive strategies for this complication.

## Conclusions

Anterior arch fracture following C1 posterior arch resection is a rare but clinically important complication that may result in progressive craniocervical instability due to disruption of the atlas ring structure.

The present case expands the limited literature by demonstrating one of the earliest reported postoperative anterior arch fractures following unilateral C1 hemilaminectomy, with symptom onset occurring on postoperative day 6. Although the underlying mechanism remains speculative, the fracture pattern observed in this case raises the possibility that asymmetric biomechanical loading after unilateral decompression, together with bone fragility associated with advanced age and concomitant multilevel laminoplasty, may have contributed to fracture development. Further biomechanical and clinical studies are warranted to clarify this mechanism.

When patients develop new-onset headache or occipital pain after C1 posterior arch resection, clinicians should maintain a low threshold for considering anterior arch fracture, and cervical CT should be considered in addition to head CT, particularly when intracranial imaging is unrevealing. In patients with progressive fracture displacement or lateral mass instability despite external immobilization, rigid internal fixation, including occiput-to-C2 fusion when appropriate, may be considered to achieve stable fixation.
